# The Co-Evolution Aspects of the Biogeochemical Role of Phytoplankton in Aquatic Ecosystems: A Review

**DOI:** 10.3390/biology12010092

**Published:** 2023-01-06

**Authors:** Nikolay V. Lobus, Maxim S. Kulikovskiy

**Affiliations:** Timiryazev Institute of Plant Physiology, Russian Academy of Sciences, Botanicheskaya St. 35, 127276 Moscow, Russia

**Keywords:** microalgae, cyanobacteria, biogeochemical cycles, essential metals, trace elements, nutrients, Redfield ratio

## Abstract

**Simple Summary:**

Currently, the development of “green” and “blue” biotechnologies, which allow for ensuring the necessary level of economic growth without additional environmental risks, is one of the keystones of technological progress in the world. Widely used in various areas of human economic activity, microalgae are the most popular objects in a wide range of living organisms, and they are used for innovative, environmentally friendly biotechnology. In our article, we consider the evolutionary aspects of the relationship of living organisms—using the examples of microalgae and cyanobacteria—with the environment. Knowledge of the habitat is one of the main tasks of such fundamental research, as well as the development of the theoretical foundations for their practical application in the national economy and the rational use of natural resources.

**Abstract:**

In freshwater and marine ecosystems, the phytoplankton community is based on microalgae and cyanobacteria, which include phylogenetically very diverse groups of oxygenic photoautotrophs. In the process of evolution, they developed a wide range of bio(geo)chemical adaptations that allow them to effectively use solar radiation, CO_2_, and nutrients, as well as major and trace elements, to form O_2_ and organic compounds with a high chemical bond energy. The inclusion of chemical elements in the key processes of energy and plastic metabolism in the cell is determined by redox conditions and the abundance and metabolic availability of elements in the paleoenvironment. Geochemical evolution, which proceeded simultaneously with the evolution of biosystems, contributed to an increase in the number of metals and trace elements acting as cofactors of enzymes involved in metabolism and maintaining homeostasis in the first photoautotrophs. The diversity of metal-containing enzymes and the adaptive ability to replace one element with another without losing the functional properties of enzymes ensured the high ecological plasticity of species and allowed microalgae and cyanobacteria to successfully colonize a wide variety of habitats. In this review, we consider the main aspects of the modern concepts of the biogeochemical evolution of aquatic ecosystems and the role of some metals in the main bioenergetic processes in photosynthetic prokaryotes and eukaryotes. We present generalized data on the efficiency of the assimilation of key nutrients by phytoplankton and their importance in the cycle of carbon, silicon, nitrogen, phosphorus, sulfur, and iron. This article presents modern views on the evolutionary prerequisites for the formation of elemental signatures in different systematic groups of microalgae, as well as the possibility of using the stoichiometric ratio in the study of biological and geochemical processes in aquatic ecosystems.

## 1. Introduction

Phytoplankton include oxygenic photoautotrophs, a phylogenetically very diverse group with a complex evolutionary history lasting for approximately 2.5–2.7 billion years. Photoautotrophs were certainly preceded by various forms of anoxygenic photosynthetic bacteria [1,2]. Despite all the diversity, phytoplankton split into evolutionarily separate functional groups, including one main group of prokaryotes (cyanobacteria, formerly referred to as blue-green algae) and a number of eukaryote groups (diatoms, cryptophytes, haptophytes, green and chrysophyte algae, etc.) [3,4]. In freshwater and marine ecosystems, microalgae and cyanobacteria form the basis of phytoplankton communities; they assimilate CO_2_ directly and produce energy-rich organic matter (OM). High species diversity and ecological flexibility allow microalgae and cyanobacteria to populate various, including extreme, habitats [5,6,7].

Being an integral component of aquatic ecosystems, microalgae and cyanobacteria play a key role in the biogeochemical cycle of major and trace elements [8,9,10,11]. They control the abundance, bioavailability, and distribution of chemical elements in water bodies and ensure their efficient accumulation and transfer along the food web; they also profoundly affect the sedimentation rates of elements as a part of the detrital component of sedimentary matter [12,13,14,15]. Along their lifespan, microalgae and cyanoprokaryotes form several alternative pathways for the migration of major and trace elements associated with their cells; each way is characterized by unique biological, biogeochemical, and geological consequences [16,17,18].

Studies of the chemical composition of microalgae and assessment of their role in the cycle of major and trace elements in the aquatic environment have a long history [19,20,21]. The obtained knowledge has contributed much to the emerging of a number of new scientific topics. Nowadays, some of them are widely used in the development of “green” and “blue” biotechnologies for solving emerging issues of anthropogenic environmental pollution [22]. In this review, we focus on the co-evolutionary aspect of the functioning of biological and geochemical cycles in aquatic ecosystems, the biochemical role of some chemical elements in the life of microalgae and cyanobacteria, and modern concepts of their stoichiometry.

## 2. Biogeochemical Evolution

The biogeochemical structure of any ecosystem and the biosphere in general is the result of the co-evolution of biological and geochemical processes that lasted billions of years on Earth [23,24]. As a result, first prokaryotes and then eukaryotes incorporated trace elements as indispensable cofactors for many biochemical and physiological processes occurring in the cell [9,25,26,27]. Depending on their chemical properties, prevalence, and availability in certain environments, chemical elements increasingly acquired various metabolic functions. As a result, accumulation, transport, and transfer of energy through redox reactions, catalyzed by metal ions or metal-containing enzymes, have become key functions for all living organisms inhabiting Earth at present. This ensured the key role of metals in many processes of energy metabolism and, subsequently, constructive metabolism in the cells of both prokaryotes and eukaryotes [28]. The evolution of photosynthesis and the saturation of the atmosphere and ocean with oxygen changed the geochemical mobility and availability of many major and trace elements fundamentally [21,29,30]. Oxygen-producing photosynthesis started a new era in the co-evolution of biological and geochemical processes, developing a variety of life forms on Earth, contributing to the forming mineral deposits, and functioning of cycles of chemical elements in the biosphere [1,27,31]. The stock and stoichiometric ratio of elements in the cells of modern cyanobacteria and microalgae are directly related to the conditions in which the organism has been evolving [23,24,32]. At the same time, the evolutionarily evolved and stabilized role of metals in enzymes’ functioning and proteins’ folding, as well as in the operating of the cell signaling systems, is considered as one of the main vectors for the development of biological diversity, both at the molecular and species levels of organization [26,33].

The result of the co-evolution of the biological and geochemical processes of freshwater and marine ecosystems is their modern “biogeochemical picture”, which includes a close relationship between the diversity of all life forms and the chemical composition of the habitat [30]. On the one hand, the abundance and availability of many major and trace elements in aquatic ecosystems have become closely related to large-scale biological cycles of photoautotrophs. On the other hand, the chemical composition of the environment controls the growth and development of communities of primary producers, regulating their taxonomic structure [21,23,34,35,36].

## 3. Physiological Role of Some Metals in Microalgae and Cyanobacteria

During their biogeochemical evolution, photoautotrophs developed a set of biochemical adaptations that allowed them to use major elements (including inorganic carbon), trace elements, and solar energy to synthesize a wide range of organic compounds [30,32]. Metals occupy a central place in the group of trace elements; they are included in the active centers of enzymes catalyzing certain processes of energy and constructive metabolism (Table 1). In nature, the content of metals in microalgae and cyanobacteria is largely predetermined by the biochemical needs of a cell at different stages of its cycle [20]. Metal ions are actively involved in the assimilation of carbon and nutrients, as well as in chlorophyll synthesis, electron transport chains of respiration and photosynthesis, and the protection of the body from reactive oxygen species; they also help to control the synthesis of DNA and RNA, the cell cycle, and many other processes [37,38,39].

Various biochemical functions were discovered and described for a large number of chemical elements in the late 20th–early 21st centuries [40]. However, it is believed that especially Fe, Zn, Mn, Cu, Co, and Ni, the most common in nature, played a key role in the biological evolution in general and in the evolution of photobiological systems and algae in particular [27,29,35,39,41].

Iron is the fourth most abundant element in Earth’s crust. Evolutionarily, it has become quantitatively the most important element involved in thylakoid-mediated reactions in all oxygenic organisms [39]. The electron flow associated with the operation of photosystems I and II (PS I and PS II) requires ~22 Fe atoms. Cytochrome *c_6_* (1 Fe atom) is the only redox catalyst binding the cytochrome *b_6_-f* complex (5 Fe atoms) with PSI (12 Fe atoms) in most algae [42].

Nitrogenase, the enzyme catalyzing nitrogen fixation in free-living and symbiotic cyanobacteria, requires 38 Fe atoms per 1 holoenzyme complex [43,44]. Diazotrophs need a significant amount of Fe, because they act as a nitrogenase cofactor. Therefore, the content of Fe in the aquatic environment and its bioavailability for diazotrophs are important for the functioning of both aquatic and global biogeochemical nitrogen cycles [45,46].

Iron, copper, and zinc are parts of the enzymes removing reactive oxygen species. These enzymes are ascorbate peroxidase (Fe) and iron– (cyanobacteria and most algae) and copper–zinc (some algae and higher plants) superoxide dismutase [32]. Fe, Zn, and Cu are also involved in a variety of cellular functions. Zinc is necessary for the correct work of carbonic anhydrase involved in the supply of CO_2_ to ribulose-1,5-bisphosphate carboxylase/oxygenase (RuBisCo) [37,39]. At the same time, RuBisCo requires Mg^2+^ ions to operate; they are located in the active center and contribute to the binding of CO_2_ with lysine residue, forming carbamate. This complex of enzymes is one of the most important in nature because it plays a key role in the main mechanism for the entry of inorganic carbon into the biological cycle and the conversion of CO_2_ into energy-rich molecules of OM [47,48]. Zn ranks second after Fe in content and participation in chemical, structural, and regulatory processes in the cell [49]. According to metal/proteomic analyses, the need for Fe decreased evolutionarily, along with an increase in Zn use in biosystems. Now, Zn performs functions that were once supported by other metal ions [50,51].

The role of other metals In cellular biochemistry is somewhat more specific and limited. Mn is most actively involved in the O_2_-producing complex of PSII; the Mn-bearing form of superoxide dismutase (SOD) is also common in diatoms. Based on metal cofactors in algae, four metal-bearing forms of SOD have been found, containing Fe, Mn, Cu + Zn, or Ni ions. Orthologues of all SOD metal-bearing forms are present in oxygenic photoautotrophs, while some species of microalgae can use more than one metal as a cofactor [52]. For example, when the Fe content is limited, some diatoms may use Mn instead of Fe in SOD [53]. Cu is used in both photosynthetic and respiratory electron transport chains, as well as in the Cu-Zn form of superoxide dismutase. In freshwater and marine microalgae, Cu also plays an important role in the transmembrane uptake of Fe [54,55]. Ni is associated primarily with urease and the Ni-bearing form of superoxide dismutase, while Co is a component of vitamin B_12_ [20].

Evolutionary transformations of enzymatic systems in photoautotrophs, associated with changes in the redox conditions, content, and bioavailability of elements in the paleoenvironment, contributed to the search for and use of alternative sources of essential metals [24,30,33,39]. As a result, microalgae and cyanoprokaryotes are able to replace some elements with others in various biochemical processes [21,23,32]. For example, Co and Cd can replace Zn in the carbonic anhydrase of some marine diatoms and coccolithophores [56,57]. This replacement has an adaptive value and makes it possible to maintain the growth and development of algae at Zn-limitation in the aquatic environment [20,25]. At the same time, the incorporation of Cd into the active center of carbonic anhydrase in some microalgae is the only known case of the biological (essential) function of the metal [58]. Oceanic diatoms that evolved in Fe-poor waters have replaced the Fe-bearing enzyme cytochrome *c_6_* in PS II by plastocyanin, the Cu-bearing enzyme. As a result, selection pressure has led to the use of a Cu-bearing protein for photosynthesis in *Thalassiosira oceanica*, the oceanic diatom species. This biochemical switch reduces the demand for Fe and increases that for Cu, which is relatively abundant in the open ocean [59,60]. Under conditions of Fe-limitation, other species of oceanic microalgae and cyanobacteria are able to replace the Fe-S ferredoxin in PS I by the metal-free protein flavodaxin or to reduce the content of Fe-rich PS I compared to PS II [39,60,61].

These biochemical adaptations, as well as some others, developed in microalgae and cyanobacteria and are associated with changes in the content and availability of elements in the aquatic environment, and they are a direct consequence of the geochemical transformations that took place in different geological epochs during biosphere formation and evolution. This provided a wide ecological plasticity of species and allowed them to master a variety of habitats effectively [21,23,29,40].

## 4. Phytoplankton and the Biogeochemical Cycle of Some Nutrients

### 4.1. Carbon

In freshwater and marine ecosystems, the functioning of the biological carbon pump (BCP) contributes to the transition of dissolved inorganic carbon compounds into solid carbonates and OM. This process ensures the interconnection of the two branches (i.e., carbonate and organic) of the complex biogeochemical cycle of carbon on the Earth [62,63].

Microalgae and cyanobacteria are major primary producers in aquatic ecosystems and play a key role in the functioning of the BCP that assimilates CO_2_ from the atmosphere [6]. Annually, they account for approximately half of the global primary production of OM in the biosphere. The efficiency of the BCP is a function of phytoplankton physiology and community structure, which are in turn governed by the physical and chemical conditions of the aquatic ecosystems [64]. The BCP in aquatic ecosystems is hypothesized to have played a significant role in atmospheric CO_2_ fluctuations during past glacial-interglacial periods, and it also responds to contemporary variations in the climate. Understanding the response of microalgae and cyanobacteria, the key mediators of the BCP, to changing environmental conditions is a prerequisite to predict future atmospheric concentrations of CO_2_ and climate dynamics [65,66].

The stock of phytogenic carbon in aquatic ecosystems includes a vast number of various components of OM of different molecular weights, ranging from small molecules, such as amino acids, organic acids, sugars, and nucleotides, to high molecular weight polymers [63,67,68]. Most of the organic carbon (OC) generated in the photic zone of freshwater and marine ecosystems is recycled by heterotrophic biota in the water masses. Only a small part of it reaches the bottom and accumulates. The processes of CO_2_ assimilation and mineralization of OM in the water column and at the surface of bottom sediments, as well as the sedimentation rate, predetermine both the temporal and quantitative parameters of CO_2_ removal from the atmosphere [62].

The photosynthetic activity of phytoplankton in the World Ocean produces ~100 × 10^15^ g OC per year in the form of gross primary production, of which ~60 × 10^15^ g OC is accounted annually for net primary production [62,69,70]. The primary sources of phytogenic carbon in the ocean also include the production of microphytobenthos (~14 × 10^12^ g OC per year), created by diatoms living in bottom sediments and by ice microflora (~17 × 10^12^ g OC per year). It should be noted that the contribution of microphytobenthos, represented by multicellular algae and higher plants, to the OC balance is only ~0.6 × 10^15^ g year [62].

Nowadays, ~80% of the primary production in the ocean is provided by diatoms; thus, the key role of this group of microalgae in the biogeochemical carbon cycle is undoubtful [60,71,72]. The mineralization of OC to CO_2_ proceeds mainly in the water column (~97%), as well as in the upper layer of bottom sediments (~2.6%). Annually, ~250 × 10^12^ g OC sinks to the bottom, which is ~0.4% of the total primary production in the World Ocean [63,64].

The inorganic branch of the carbon cycle operates due to the ability of living organisms to synthesize solid carbonates. Living organisms assimilate ionic forms of carbon dioxide from solution and convert them into biogenic calcite and aragonite. The biosynthesis of carbonates is an ancient process that originated in prokaryotes approximately 3.5 billion years ago. The cyanobacteria communities were developing bacterial mats and stromatolites of varying thickness. However, under modern biogeochemical environmental conditions, they have survived only in the form of thin films in the coral reef ecosystem and have lost their leading role in the accumulation of CaCO_3_ [62]. Currently, coccolithophorides are one of the main groups of organisms involved in the synthesis of biogenic carbonates. These are marine unicellular planktonic haptophyte microalgae, which account for ~1 up to 40% (when blooming) of the primary OC production and ~18–20% of the total carbonate biosynthesis in the ocean (~1.4 × 10^15^ g of inorganic carbon per year). Approximately 50% of carbonates synthesized by coccolithophores are dissolved in the water column, replenishing the stock of dissolved inorganic forms of carbon in the ocean. This group of planktonic microalgae makes a significant contribution to the functioning of two branches of the biogeochemical carbon cycle and to the accumulation of CaCO_3_ in bottom sediments [73,74].

The climatic zone is one of the main factors in the primary productivity of phytoplankton in freshwater ecosystems of rivers and lakes. Despite significant complexity in assessing the total phytoplankton production in freshwaters, ~70–72 × 10^12^ g OC is deposited in the bottom sediments of rivers and 0.65 × 10^15^ g OC in lakes and artificial reservoirs [69].

Microalgae and cyanobacteria are responsible for the sequestration of most of the CO_2_ by primary producers for various biosynthetic processes and the corresponding amount of organic and inorganic forms of carbon deposited in the sediments of freshwater and marine ecosystems; thus, they play a key role in the global biogeochemical carbon cycle in the biosphere and influence much of Earth’s climate [62,64,66].

### 4.2. Silicon

Silicon is the second most abundant element in Earth’s crust. It plays a key role in biological processes, being an important nutritional and structural component for many aquatic and terrestrial organisms [75]. The mobilization of Si from primary minerals (formation of silicic acid (dSi; Si(OH)_4_)), dSi migration in water, and dSi assimilation by silicifiers (formation and subsequent precipitation of biogenic silica (bSi; SiO_2_ × nH_2_O)) are key links in the biogeochemical cycle of Si in the biosphere [5,76]. Phototrophic silicifiers, such as diatoms, globally consume huge amounts of Si, along with nitrogen, phosphorus, and inorganic carbon, combining the biogenic migration of these elements and contributing to the sequestration of atmospheric CO_2_ in freshwater and marine ecosystems [77,78]. In aquatic ecosystems, the Si biogeochemical cycle is most closely related to the carbon cycle due to the fact of its influence on the growth and development of diatoms. Their dense siliceous cell walls promote the sinking of solid particles (and all of their organic carbon and nutrients). Therefore, diatoms are believed to be the main organisms responsible for the low levels of dSi observed at the ocean surface and for the transport of the mineral silica to depths [79,80]. Due to the higher content of dSi in fresh waters, its complete depletion in the photic layer of rivers and lakes is less common. However, during mass diatom blooms, the concentration of dSi in the active layer of freshwater ecosystems may decrease sharply [76,81].

The data available in the literature indicate significant differences in the estimates of the content of bSi in the cells of freshwater and marine diatoms [18]. First, this is due to the difficulty of collecting phytoplankton samples that do not contain microimpurities of terrigenous matter [18,82], as well as due to the fact of significant species differences [83,84] and attempts to extrapolate the results of laboratory studies of certain species to natural communities of microalgae [85,86]. A general pattern may be traced when comparing the ecological aspects of the accumulation of bSi in diatoms inhabiting freshwater and marine ecosystems. On average, marine diatoms contain ~10 times less bSi than freshwater diatoms [18,81,87]. These differences may be related to the evolutionary adaptation of diatoms to the abundance and bioavailability of dSi in freshwater and marine waters, as well as to the strategies for maintaining cell buoyancy in water of different densities and protecting them from grazing pressure [80,81,87].

The photosynthetic activity of diatoms in the World Ocean results in assimilating a large amount of dSi. The latest estimate of the global gross marine pelagic bSi production amounted to 6.74 ± 1.12 × 10^15^ g Si per year [88]. Currently, based on new data from 49 field studies and the results of 18 mathematical models, the global bSi production in the pelagic zone, formed mainly by diatoms, has been updated, amounting to 7.16 ± 1.45 × 10^15^ g Si per year [78], which is close to the previous estimates [88]. Globally, ~50–60% planktonic bSi is dissolved and recycled in the upper photic layer of the ocean. About 40% of bSi is mineralized in the deep layers of the water column and in the upper layer of bottom sediments, returning to the biogenic cycle due to marine and oceanic upwellings. Long-term stock of plankton bSi in ocean sediments is estimated as 0.15 ± 0.03–0.26 ± 0.05 × 10^15^ g Si per year, which is ~2.0–3.5% of the global pelagic production of bSi [78,88,89,90,91].

The discovery of Si accumulation by abundant cosmopolitan picocyanobacteria *Synechococcus* identified new and previously unknown aspects of the biogeochemical cycle of this nutrient [92]. The physiological role of Si in *Synechococcus* has not yet been established, but experiments with a number of strains cultivated in the laboratory evidence that Si uptake may be related to phosphate assimilation, because the attenuation of P-stress temporarily reduces the uptake of Si. It is suggested that the accumulation of Si by *Synechococcus* occurs unintentionally [93]. This is confirmed by the study of the chemical forms of Si in the cells of several strains of *Synechococcus.* In particular, Si is presented by a silicate oxyhydroxide or aqueous oxide in their cells, and these compounds differ spectrally from the amorphous bSi accumulated by diatoms [94]. The preliminary estimate of the contribution of *Synechococcus* to the accumulation of silicon in the ocean is less than 0.56 × 10^15^ g Si per year [78]. It should be noted that this value falls within the error of estimates of the diatom contribution to Si accumulation. However, it has been suggested that *Synechococcus* may have a previously unrecognized effect on ocean Si cycling, especially in nutrient-poor open ocean waters [92].

There are significantly fewer data on estimates of the annual bSi production in freshwater ecosystems. Regard must be paid to the freshwater biological production of bSi, which is associated with the assimilation of dSi by diatoms; during this process, the chemical form of Si, originating from land and coming to the World Ocean with river runoff, is modified [89]. The global contribution of bSi supplied by river runoff into marine ecosystems is estimated as 29.5 ± 5.6 × 10^12^ g Si per year. If considering a global average concentration of dSi in river waters as 150 µmol L^−1^, ~16% of gross Si, supplied by river runoff to the World Ocean, it comes as bSi synthesized by freshwater diatoms. Most of this bSi will be remobilized by dissolving into the marine environment. These data show that the contribution of freshwater bSi, carried as particulate organic matter by rivers, is an important component of the Si balance in the World Ocean that was not previously taken into account [95].

### 4.3. Nitrogen

In both freshwater and marine ecosystems, the nitrogen cycle controls water productivity. Unlike other major elements (for example, Si), it is highly demanded by absolutely all groups of microalgae, because N is necessary for cell growth and development, often being the main limiting nutrient in the environment [96,97]. The nitrogen cycle is driven by complex biogeochemical transformations, including N fixation, denitrification, assimilation, and microorganism-mediated anaerobic oxidation of ammonia. Genomic studies have revealed the diversity of N metabolism strategies in microalgae and cyanobacteria [98]. The nitrogen cycle in freshwater and marine ecosystems is an integral feature of their functioning. It plays a key role in ecosystem responses to global environmental changes associated with an anthropogenic impact and climate fluctuations [8,99].

The many oxidation states of N in the aquatic environment predetermine a large number of redox reactions that convert one chemical form into another. In modern environmental conditions, all of the main processes of the biogeochemical N cycle, mediated by the vital activity of phytoplankton, are associated with the assimilation of NO_3_^−^, NO_2_^−^, and NH_4_^+^ and with the biological fixation of N_2_ [100]. The uptake of NO_3_^−^ and NH_4_^+^ by microalgae and the conversion of inorganic forms of N into organic forms dominate quantitatively in the N cycle in the aquatic environment. It is believed that NH_4_^+^ is the preferred source of N for phytoplankton, because its assimilation does not involve redox reactions and requires little energy [101]. On the contrary, the assimilation of NO_3_^−^ involves the reduction of nitrogen from the oxidation state +V to −III (1) and requires a significant amount of energy to transfer eight electrons. After moving through the plasmalemma (which is an energy-dependent process), the assimilation of NO_3_^−^ requires chemical reduction to NH_4_^+^. This process has two stages, and it is mediated by two enzymes: nitrate reductase (1) and nitrite reductase (2) [102]:

NO_3_^−^ + 2e^−^ + 2H^+^ → NO_2_^−^ + H_2_O (1)

NO_2_^−^ + 6e^−^ + 8H^+^ → NH_4_^+^ + 2H_2_O (2)

The general stoichiometry for the reduction of nitrate to ammonium by microalgae and cyanobacteria may be presented as:

NO_3_^−^ + 8e^−^ + 10H^+^ → NH_4_^+^ + 3H_2_O (3)

As NO_3_^−^ in the aquatic environment is generally much more abundant than NH_4_^+^, most species of microalgae and cyanobacteria have the enzymes required to affect this reduction (i.e., nitrate reductase), with a few notable exceptions. *Prochlorococcus* and some strains of *Synechococcus* are the most studied organisms that are not able to use NO_3_^−^ as a source of N [103]. All groups of microalgae that are able to use NO_3_^−^ also have nitrite reductase and can assimilate NO_2_^−^ (as a source of N). However, NO_2_^−^ is usually only a very insignificant source of N for microalgae, because its concentration in the environment is an order of magnitude lower than the concentration of NO_3_^−^ or NH_4_^+^ [104].

The biological fixation of N by diazotrophs has a central place in the biogeochemical cycle. Nonreactive atmospheric N_2_ is reduced to NH_4_^+^ compounds (4 and 5) and then assimilated and converted into organic nitrogen [44,105].

N_2_ + 8H^+^ + 8e^−^ + 16ATP → 2NH_3_ + H_2_ + 16ADP + 16P_i_ (4)

NH_3_ + H_2_O ⇄ NH_4_^+^ + OH^−^ (5)

In aquatic ecosystems, predominantly in areas where other biologically available forms of nitrogen are limited or depleted, biological nitrogen fixation (BNF) is performed by cyanobacteria [106]. Cyanobacteria are both free-living organisms and form a large number of symbiotic associations [44]. They are metabolically versatile, flexible, and sensitive. The oxygenic photosynthesis with CO_2_ fixation is a usual growth mode for cyanobacteria. However, many species can also absorb and assimilate various organic compounds, such as carbohydrates and amino acids [46]. They can use nitrates, nitrites, ammonium, amino acids, and urea as a source of nitrogen. The process of N_2_ fixing is energy consuming. It takes 16 ATP molecules to assimilate one N_2_ molecule (4). In cyanobacteria, this need is covered by photosynthesis. In the aquatic environment, the rate of the BNF is high only when cyanobacteria constitute the main part of phytoplankton biomass [106,107].

In freshwater ecosystems, N_2_ fixation by plankton is closely related to the trophic status of a water body. Using lakes as an example, it is shown that N_2_ fixation by plankton tends to be low in oligotrophic and mesotrophic water bodies (≪0.1 g N m^−2^ year^−1^), but it is often high in eutrophic ones (~0.2–9.2 g N m^−2^ year^−1^). In the shallow waters of rivers and lakes, BNF in cyanobacterial mats has the greatest importance. The efficiency of N_2_ fixation varies over a wide range and may reach very high values (1.3–76.0 g N m^−2^ year^−1^) [108]. Typically, bacterial mats cover only a small area of an ecosystem, which limits their contribution to the nitrogen supply balance. In general, the significance of planktonic N_2_ fixation in freshwater ecosystems remains a highly debatable issue. For example, BNF appears to be unimportant as a source of N for most oligotrophic and mesotrophic lakes (typically <1% of total N input), but it accounts for 6 to 82% of the total N input in eutrophic lakes [106,107,108].

In the open ocean, the most prominent and best studied diazotroph is *Trichodesmium* [44,45]. These are planktonic, filamentous, nonheterocystous cyanobacteria that fix atmospheric N_2_ and convert it into NH_4_^+^, which is used by this cyanobacteria and by other microalgae surrounding its cells [109]. N_2_ fixation in *Trichodesmium* is unique among diazotrophs, because this process occurs in them simultaneously with the photosynthetic production of O_2_. In other cyanobacteria, N_2_ and CO_2_ reduction processes are separated either in space (using heterocysts to protect the sensitive nitrogenase enzyme from oxygen) or in time [110]. Some other diazotrophic cyanobacteria in the ocean are symbionts. The best-known heterocyst species is *Richelia* sp., which lives intracellularly in large planktonic diatoms, such as *Rhizosolenia* and *Hemiaulus* [111]. Many single-cell symbiotic cyanobacteria have also been found in dinophytes and radiolarians. The ecological role of all symbiotic diazotrophs is to provide the host cells with biologically available forms of nitrogen [44]. Despite the wide taxonomic diversity of aquatic nitrogen-fixing cynanobacteria, *Trichodesmium* appears to be the dominant group of diazotrophs globally. It annually accounts for approximately half of the BNF performed by cyanobacteria in the ocean [109].

The magnitude of the assimilation of dissolved inorganic forms of nitrogen by phytoplankton and the fixation of N_2_ in the aquatic environment has remained the subject of intensive research, discussion, and controversy since the 1990s [104,106,107,108,112]. At the same time, the calculations of the global BNF in marine and oceanic waters are more stable, estimated as 125 × 10^12^ g N per year [113], in the range of 60—200 × 10^12^ g N per year [114]. Other BNF calculations have reported 140—145 × 10^12^ g N per year [115,116]. The recent estimates of global marine BNF are approximately 140 ± 50 × 10^12^ g N per year. At present, this value is practically accepted and widely used in calculations of the element balance and in mathematical models [112]. It should be noted that the total contribution of BNF by the planktonic diazotroph *Trichodesmium*, as mentioned above, is approximately 60—80 × 10^12^ g N per year [109].

Currently, the regional and global nitrogen budgets are significantly affected by the dramatic increase in the input of anthropogenic bioavailable N in the form of synthetic fertilizers and agricultural, industrial, and household wastes, as well as nitrogen emissions, both in oxides and reduced forms. The eutrophication of freshwater and marine ecosystems and a sharp decrease in water quality are the consequences of disruption of the biogeochemical cycle of N in the biosphere [109].

### 4.4. Phosphorus

Phosphorus (P) is the eleventh most abundant chemical element in Earth’s crust; it is present in relatively small amounts (~0.08–0.09% by mass). Once P is released (i.e., mobilized) from minerals during weathering, it is rapidly sequestered into a series of more stable phases, which limits its daily availability to living organisms [117,118]. Despite this, during biogeochemical evolution and selection, P has become central to many indispensable bioenergetic processes, including photosynthesis and respiration [30,119,120]. Phosphorus takes an active part in the synthesis of proteins, enzymes, co-enzymes, and phospholipids. It is a key component of nucleic acids (DNA and RNA), playing a critical role in the storage, replication, and transcription of genetic information. Phosphorus is part of the biochemical components of different energy levels (adenosine tri-, bi-, and monophosphate). The relative amount of the latter characterizes the metabolic activity of cells [21,121].

In aquatic ecosystems, microalgae and cyanobacteria occupy a central place in the biogeochemical cycle of this element [122,123]. Phosphorus, like nitrogen, is an essential nutrient; its deficiency limits the growth and development of phytoplankton, thus reducing the ecosystem productivity. However, the current paradigms of the P-limitation of primary production in freshwater, estuarine, and marine environments are completely different. A review of in vivo and in situ data analyzing the factors limiting phytoplankton development concludes that P-limiting in a freshwater environment may be demonstrated at several hierarchical levels of system complexity, from algae cultures to entire lakes [97]. However, the role of P-limitation in regulating the production of marine ecosystems remains a pending issue. There is a strong opinion that, unlike N, P limits the global ocean primary production on geological time scales, i.e., for periods exceeding 1000 years [97,124]. The theory behind this P-limitation of primary production in marine ecosystems is relatively simple. In the ocean, the long-term N needs of phytoplankton can be met by the process of N_2_ fixation. As the stock of N_2_ in the atmosphere is very large, the activity of planktonic diazotrophs is able to compensate for the loss of N in the photic layer of the ocean due to the N_2_ fixation and the formation of biologically available NH_4_^+^ [45,109]. Given the long residence time of P in oceanic waters, microalgae will eventually be limited to other nutrients, such as N, Si, Fe, or trace elements [117,125,126]. This widespread opinion has led to the study of the marine link of the biogeochemical P cycle, which was focused mainly on the identification and balance of oceanic sources of P input and removal [97,125,127].

In aquatic ecosystems, the P content in the euphotic zone, where phytoplankton grows, is predetermined by the balance between the local processes of input and the removal of this element. Microalgae and cyanobacteria effectively assimilate the bioavailable stock of P represented by dissolved inorganic phosphorus (DIP = HPO_4_^2−^ + PO_4_^3−^) and dissolved organic phosphorus (DOP). Microalgae preferentially use DIP, because it can be taken up directly and assimilated to support cell metabolism and growth, while DOP is usually converted into DIP prior to its metabolic assimilation. The last requires large energy costs [119]. However, when the external DIP stock is depleted, the growth of microalgae and cyanoprokaryotes often depends on their ability to utilize more abundant DOP stock by enzymatic hydrolysis resulting in DIP [123].

The efficiency of P assimilation by microalgae and cyanobacteria is so high that its concentration in water may decrease to an analytical zero during phytoplankton blooms. Under the conditions of significant spatial and temporal variability of the P content in water, microalgae are capable of intracellular accumulation of excess PO_4_^3−^, which does not require immediate biochemical transformation [123]. The accumulation of P maintains the metabolic needs of cells in this element and ensures high growth rates of the microalgae population for several generations under subsequent conditions of low P content in the environment [128,129]. The formation of polyphosphate, which consists of a large number of phosphate residues linked by high-energy phosphoanhydride bonds, is the main known mechanism of intracellular P accumulation in microalgae [130]. In addition to the assimilation by cellular biomolecules, PO_4_^3−^ may also be adsorbed directly on the surface of microalgae cells. The amount of adsorbed P varies from 15 to 90% of its total content in the cell [131]. Adsorbed PO_4_^3−^ may play an important but poorly understood role in intracellular DIP uptake, especially in the environment, where the DIP content fluctuates significantly and is rich in Mn and Fe. If microalgae cells are capable of using mechanisms (for example, phagotrophy) to absorb adsorbed PO_4_^3−^, then its adsorption accumulation on the cell wall may be considered as an external reservoir for P storing [132]. The intracellular accumulation of P in the form of polyphosphates and its sorption on the cell surface are of great adaptive importance for microalgae.

The biological assimilation of DIP, its biosorption, and subsequent removal of P from the euphotic zone of freshwater and marine ecosystems are among the key aspects of the water cycle of the element’s biogeochemical migration [123,125]. The sedimentation of the biogenic detritus and its partial microbiological mineralization enrich the deep layers of the water column with DIP, which can again enter the euphotic layer through vertical mixing or upwelling, provided by hydrodynamics. Part of this organic material reaches the bottom in a biochemically transformed form [70] and accumulates in sediments. This leads to the removal of P from the global biogeochemical cycle [125].

At present, our understanding of the magnitude of the assimilation of P and other nutrients by microalgae remains uncertain. This is due to the differences in the primary production estimates in different water bodies. This issue has been most studied for marine ecosystems. It is believed that approximately ~1.2 × 10^15^ g P per year is assimilated globally via primary production in the World Ocean [133]. At the same time, the amount of biogenic phosphorus buried in the sediments of the World Ocean is estimated as ~0.34–1.26 × 10^12^ g P per year [125].

The phosphorus cycle is strongly influenced by the eutrophication of lakes and the burial of large amounts of planktonogenic OM rich in P. However, assessing the P accumulated in the sediments of freshwater basins and the role of the latter in the removal of P from the local and global biogeochemical circulations remain currently undeveloped issues [134,135].

### 4.5. Sulfur

Sulfur (S) is the fifteenth most abundant chemical element in Earth’s crust and the sixth most abundant in the aquatic environment. In natural waters saturated with O_2_, sulfur is presented mainly as SO_4_^2−^ [136]. As for most photosynthetic organisms, SO_4_^2−^ is the most bioavailable form of S for microalgae. Sulfur is essential for maintaining cellular homeostasis; it is part of amino acids (cysteine and methionine), proteins, enzymes, and vitamins (B_1_ and biotin). Its deficiency has a strong effect on photosynthesis, the assimilation of carbon and nitrogen, the functioning of cell defense mechanisms, and, in general, on the processes of constructive and energy metabolism [137,138]. Phytoplankton assimilates approximately 1.32 × 10^15^ g S per year [139].

In microalgae and cyanobacteria, sulfur is absorbed as SO_4_^2−^, and then it is reduced to sulfide (S^2−^) in chloroplasts [140]. Further, practically all reduced sulfur is involved in the synthesis of cysteine, which directly or indirectly serves as a precursor of compounds containing reduced sulfur [141]. In oxygenic photoautotrophs, the position of cysteine biosynthesis between the assimilation of inorganic sulfate and the metabolization of organic sulfide makes it one of the key processes for incorporating S into biogenic migration [142].

In freshwater and marine ecosystems, the availability of SO_4_^2−^ may differ significantly, which predetermines the noticeable differences in the strategies used by microalgae for its assimilation [137]. In the ocean, the concentration of SO_4_^2−^ is never limited, remaining constantly very high. However, fresh waters are characterized by a fairly wide range of both daily and seasonal changes in the SO_4_^2−^ content. In some cases, the SO_4_^2−^ concentration may be very low, acting as a limiting factor [139,140]. In freshwater ecosystems, the sulfur limitation left an evolutionary imprint on the proteome of photosynthetic organisms. Cyanobacteria *Calothrix* sp., deficient in S, express light-harvesting phycobilins with lower levels of cysteine and methionine compared to that expressed in S-rich cells [143]. Subsequent studies show evidence of similar proteomic responses in living organisms at different trophic levels related to prokaryotes and eukaryotes, which are prone to sulfur deficiency [144].

In marine ecosystems, dimethylsulphoniopropionate (DMSP) formation in oxygenic photoautotrophs is an important aspect of the sulfur biogenic cycle [139,145,146]. The biosynthesis of DMSP is carried out from methionine by many species of marine microalgae (primnesiophytes, dinophytes, diatoms, chrysophytes, and prasinophytes) and some species of littoral macroalgae (chlorophytes and rhodophytes) [102,142]. This process proceeds most intensively at elevated salinity and N-limitation. In marine microalgae, DMSP accumulates in cells in large amounts and functions as an osmoregulator, buoyancy regulator, cryoprotector, and antioxidant [145,146,147]. The amount of organic S in the form of DMSP in seaweeds may reach approximately 70% of the total S content in the cell [148]. DMSP, produced by marine microalgae and higher plants, is part of their osmoregulatory system. For a long time, this process has been described exclusively for salt waters and, thus, considered insignificant for freshwater species [145,147]. However, by example of freshwater dinophyte *Peridinium gatunense*, it has been reported that its cells store a significant amount of DMSP, especially in the stationary growth phase [149].

DMSP is a precursor of biogenic dimethyl sulfide (DMS), which is formed as a result of enzymatic cleavage by DMSP-lyase. Several forms of DMSP-lyases are known and have been found in various groups of marine bacteria (seven), fungi (one), and microalgae (one) [102,146]. However, the algal enzymes responsible for the formation of DMS from DMSP still remain poorly understood, despite their critical role in the global S cycle [150]. In an aquatic environment, DMS plays an important role as a chemical signal that mediates various trophic interactions [102,151]. DMS is a gas, and it is one of the main pathways for the entry of bioavailable sulfur into the biogeochemical cycle [152]. It accounts for ~50% of the biogenic sulfur entering the atmosphere. The magnitude of the global DMS emission from the ocean has different estimates and depends on the data availability for the models used [153]. Recent calculations estimate the global flux of biogenic DMS from the ocean surface to the atmosphere as 20.12 ± 0.43 × 10^12^ g S per year [154].

The atmospheric oxidation of DMS derived from marine phytoplankton is the most common source of naturally occurring sulfate aerosol particles [152]. DMS, interacting with radicals, is oxidized and forms SO_2_, which acts as a direct precursor of aerosol SO_4_^2−^ particles [146,150]. They are able to backscatter incoming solar energy and act as cloud condensation nuclei (CCN) over the ocean. Both processes have a cooling effect on the atmosphere and, thus, affect the solar radiation balance and the Earth’s climate. This algal climate regulation is called the CLAW hypothesis; it is important for understanding the modern global biogeochemical sulfur cycle in the biosphere [139,142,155,156].

### 4.6. Iron

Iron is the fourth most abundant chemical element on Earth and an essential nutrient for all photosynthetic organisms. Microalgae and cyanobacteria, like all photoautotrophs, must maintain a Fe-rich photosynthetic electron transport chain that was probably formed in the Fe-rich reducing oceanic environment in the Proterozoic [1,24,27,33]. The bioavailability of Fe dropped sharply with the advent of photosynthesis and an increase in the O_2_ content in the environment. This led to a wide evolutionary response in various groups of phytoplankton, changing dramatically the biogeochemical cycle of Fe in the biosphere [157]. Microalgae and cyanobacteria have developed a wide range of molecular–genetic and physiological–biochemical mechanisms of adaptation to Fe deficiency in the environment [158,159,160].

Freshwater ecosystems are not deficient in dissolved Fe. However, its concentration in the open ocean is several orders of magnitude lower, even compared to coastal waters. This is reflected in the taxonomic structure of phytoplankton communities and the production of marine and oceanic ecosystems [161]. Approximately 40% of the World Ocean is characterized by a sufficient supply of nutrients (N, P, and Si), but a low content of chlorophyll (HNLC regions). The main factor limiting the growth and development of microalgae and cyanobacteria is not the total content of Fe in the environment, but the concentration of bioavailable Fe [35,162].

The ocean contains a number of different physicochemical fractions of dissolved iron (dFe), including Fe^2+^ and Fe^3+^, colloidal and inorganic Fe, and organically bounded Fe. It is widely believed that organic iron-binding ligands form complexes with more than 99% dFe in the ocean [163,164]. The distribution of dFe is predetermined by a combination of processes: the solubility of Fe^2+^ and Fe^3+^ in various redox environmental conditions, rate of chemical transformations of Fe^2+^ ⇄ Fe^3+^, rate of Fe transfer within oceanic basins, absorption and release of Fe by planktonic organisms, intensity of the input from various sources, as well as the final amount of Fe burial in bottom sediments [165].

Marine cyanobacteria, unlike microalgae, potentially have two mechanisms of Fe uptake. Its assimilation may be carried out both with the help of Fe ion membrane carriers and of siderophore systems [160]. They function through the excretion of low molecular Fe chelators. They selectively bind Fe; then, they are transported back into cells using chelate-specific transport proteins [163]. Although classical siderophores have been isolated and characterized in some species of marine cyanobacteria, generally their use as a Fe-uptake mechanism in planktonic free-living species may be limited. At the same time, cyanobacteria inhabiting the open ocean and not producing their own siderophores are likely to use siderophores produced by other organisms [164,166]. There is currently no robust evidence that siderophore systems are used by eukaryotic marine phytoplankton. Various forms of the ion membrane transfer of Fe are described both for microalgae and cyanobacteria [35,163,167].

The molecular mechanisms of Fe uptake used by oceanic phytoplankton need to be further explored. However, their efficiency is very high, because it allows microalgae and cyanobacteria to absorb Fe from the environment, where it is present in subnanomolar concentrations [158,164]. Combining various estimates of the magnitude of the primary production and the metabolic need of cells for Fe gives the overall estimation of the fixation rates of this metal by marine phytoplankton. In coastal seas, the assimilation rate is 13.5 × 10^12^ g Fe per year and in the open ocean, 3.1 × 10^12^ g Fe per year. Together, this amounts to 16.6 × 10^12^ g Fe per year for the entire World Ocean. In surface waters, primary OM is mineralized, and chemical elements are repeatedly recycled. Only an insignificant part of the OM goes into deeper waters as an export product. The contribution of phytoplankton to the export of Fe from the zone of active photosynthesis to deeper layers is approximately 3.4 × 10^12^ g Fe per year in coastal seas and 0.31 × 10^12^ g Fe per year in the open ocean. In total, ~3.71 × 10^12^ g of biogenic Fe is exported from the euphotic layer per year [165].

## 5. Multi-Element Composition and Phytoplankton Stoichiometry: Evaluation and Application Issues

Because phytoplankton lives at the interface between the abiotic and biotic components of ecosystems, it plays a key role in connecting the cycles of multiple chemical elements [5,9,18,20,21,22]. Quantitative estimates of how these biogeochemical cycles intersect are determined by the phytoplankton stoichiometry [168]. For a long time, the content and ratio of the main biogenic elements (C:N:P) in phytoplankton have been studied intensively; traditionally, it has been considered that their stoichiometry has limited variability [169]. Any discussion of phytoplankton stoichiometry should begin with the work of Alfred Redfield [170]. Based on Fleming’s work [171], he declared the average C:N:P atomic ratio in ocean phytoplankton to be 106:16:1. Redfield’s fundamental understanding of the chemistry of aquatic ecosystems did not depend on specific element ratios, per se, but rather involved an understanding of the relationship between plankton chemistry (specifically the N:P ratio) and seawater. Redfield gave a rather striking interpretation of the causal relationship of this phenomenon. He suggested that the ratio of nitrates and phosphates in the interior of the ocean was almost identical to their ratio in plankton, because plankton predetermined the chemical composition of the ocean [172]. At the time, this was a radical idea that was two decades ahead of the equally revolutionary concept of Gaia put forward by James Lovelock in 1979 [173]. The works of Redfield and his followers have made an invaluable contribution to the development of production biology, biogeochemistry of aquatic ecosystems, geochemistry of sedimentary processes, and many other scientific areas [5,38,169].

Later studies drew attention to the fact that the functioning of phytoplankton is not predetermined only by the ratio of the main biogenic elements (N:P) and their availability in the environment [174]. As shown above, a wide range of major and trace elements is required for the operation of a large number of enzymatic systems responsible for the assimilation of C, N, and P, in addition to other performed functions [35,38,39,161]. The HNLC regions of the ocean, which occupy up to 40% of its area, may serve as a striking confirmation of this phenomenon [35,162]. Chemical studies on natural phytoplankton communities evidence the multi-element nature and high variability of its composition [18]. As knowledge of phytoplankton stoichiometry expands through the inclusion of major and trace elements, the original Redfield trio, C:N:P, remains noticeably unchanged, while the relation itself takes the form [37]

(C_124_ N_16_ P_1_ S_1.3_ K_1.7_ Mg_0.56_ Ca_0.5_) × _1000_ Sr_5_ Fe_7.5_ Zn_0.8_ Cu_0.38_ Cd_0.21_ Co_0.19_ Mo_0.03_.

Although originally referred to as “average”, the Redfield ratio has sometimes been misinterpreted as a universal and constant phytoplankton stoichiometry. However, its variability in space and time and between different species has been reported [168,175,176]. Interesting results have been obtained in studies on the evolution of the element stoichiometry in microalgae that use chlorophyll *b* and chlorophyll *c* as an auxiliary photosynthetic pigment [119]. There are systematic phylogenetic differences in the two types of plastids, where the major elements’ stoichiometry (C:N:P) primarily reflects the inherited presymbiotic phenotypes of the host cells, and the trace elements’ composition reflects the differences in the acquired plastids. The differences in the stoichiometry of microalgae using different auxiliary photosynthetic plastids (chlorophyll *b* and *c*) suggest that changes in the redox conditions in the ocean have strongly influenced the evolution and selection of eukaryotic phytoplankton since the Proterozoic era [24,29,32,33,177].

Redfield’s concept became the basis for the foundations of ocean biogeochemical models for many decades and has not lost its relevance at present [8,177]. The stoichiometry of biogenic elements (C:N:P) and, later, trace elements began to be applied widely in balance calculations of the efficiency of their assimilation by phytoplankton, recycling in the water column, and burial as a part of the detrital component of sedimentary matter [88,113,125,154,165]. However, many models do not take into account sedimentation and diagenetic changes in the Redfield ratio, which makes them more vulnerable, in our opinion. Profound biochemical changes occur after a phytoplankton cell dies and becomes a detrital component of sedimentary matter [177,178]. During sedimentation, the group composition of OM (proteins, lipids, and carbohydrates) changes and, consequently, the content and ratio of the main biogenic elements (C:N:P), as well as trace elements. The P content in phytogenic detritus decreases with depth approximately three times faster than N and the N content faster than OC. Organic matter reaches the bottom of the reservoir (especially at great depths) as biochemically transformed and is depleted in biogenic elements [62,70,179]. Along with this, during the sedimentation and early diagenesis, the processes of the sorption/desorption of chemical elements take place actively on the surface of organomineral particles formed after the death of a phytoplankton cell [8,11,12,180]. Taken together, these processes significantly change the chemical composition of the initial phytogenic OM formed in the photic layer of aquatic ecosystems.

## 6. Conclusions

Bioinorganic chemistry, in a broad sense, provides unique insight into the co-evolution of life and the environment. A comprehensive study of the genetic diversity of microalgae and cyanobacteria, gene expression of metal-containing enzymes, protein folding, and bioavailability of elements in aquatic ecosystems can be useful for revealing the history of the origin and development of life on Earth. These studies formed the basis of the disciplines of biogeochemistry, geobiology, and astrobiology and gave impetus to the development of their wide practical application. The fundamental work on the bioinorganic chemistry of primary producers of aquatic ecosystems provided a theoretical basis for the development of green biotechnology in the field of controlled photobiosynthesis, production hydrobiology, ecological monitoring of aquatic ecosystems, bioremediation of water bodies, treatment of waste and technogenic waters, sequestration of CO_2_ and key major elements (N and P), as well as the search for alternative biological methods for the concentration of rare and precious metals and metalloids.

## Figures and Tables

**Table 1:** Some metalloproteins and their functions in microalgae and cyanobacteria.

Metal	Enzyme(s)	Function(s) in a Cell
Fe	Cytochromes	Electron transport during photosynthesis and respiration
	Other Fe-S proteins	
	Ferredoxin	Electron transport during photosynthesis and nitrogen fixation
	NAD(P)H/PQ-oxidoreductase	Electron transport during photosynthesis
	Nitrate and nitrite reductase	Converting nitrates to ammonia
	Chelatase	Synthesis of porphyrin and phycobiliprotein
	Nitrogenase *	Nitrogen fixation
	Catalase	Protection of the cell from reactive oxygen species
	Peroxidase	
	Superoxide dismutase	
Zn	Carbonic anhydrase	Hydration and dehydration of CO_2_
	Alkaline phosphatase	Hydrolysis of Phosphoric Acid Esters
	RNA polymerase	Replication and transcription of nucleic acids
	tRNA synthetase	tRNA synthesis
	Reverse transcriptase	Synthesis of single-stranded DNA from RNA
	Carboxypeptidase	Hydrolysis of peptide bonds
	Superoxide dismutase	Protection of the cell from reactive oxygen species
Cu	Multicopper ferroxidase	High-affinity transmembrane Fe transport
	Superoxide dismutase	Protection of the cell from reactive oxygen species
	Ascorbate oxidase	Oxidation and reduction of ascorbic acid
	Cytochrome oxidase	Mitochondrial electron transport
	Plastocyanin	Electron transport during photosynthesis
Mn	Phosphotransferases	Phosphorylation reactions
	Arginase	Hydrolysis of arginine to ornithine and urea
	Superoxide dismutase	Protection of the cell from reactive oxygen species
	O_2_-releasing enzymes	Water oxidation during photosynthesis
Mo	Nitrate reductase	Converting nitrates to ammonia
	Nitrogenase *	Nitrogen fixation
Ni	Superoxide dismutase	Protection of the cell from reactive oxygen species
	Urease	Hydrolysis of urea
Co	Vitamin B_12_	Carbon and hydrogen transfer reactions

* In free-living and symbiotic diazotrophs.

## Data Availability

The dataset is presented in this study.
